# Sanitation, hookworm, anemia, stunting, and wasting in primary school children in southern Ethiopia: Baseline results from a study in 30 schools

**DOI:** 10.1371/journal.pntd.0005948

**Published:** 2017-10-09

**Authors:** Jack E. T. Grimes, Gemechu Tadesse, Iain A. Gardiner, Elodie Yard, Yonas Wuletaw, Michael R. Templeton, Wendy E. Harrison, Lesley J. Drake

**Affiliations:** 1 Department of Civil and Environmental Engineering, South Kensington Campus, Imperial College London, London, United Kingdom; 2 Ethiopian Public Health Institute, Addis Ababa, Ethiopia; 3 Partnership for Child Development, Department of Infectious Disease Epidemiology, St Mary’s Campus, Imperial College London, London, United Kingdom; 4 Schistosomiasis Control Initiative, Department of Infectious Disease Epidemiology, St Mary’s Campus, Imperial College London, London, United Kingdom; Yale Child Health Research Center, UNITED STATES

## Abstract

**Background:**

Inadequate nutrition; neglected topical diseases; and insufficient water, sanitation, and hygiene (WASH) are interrelated problems in schools in low-income countries, but are not routinely tackled together. A recent three-year longitudinal study investigated integrated school health and nutrition approaches in 30 government primary schools in southern Ethiopia. Here, we report on baseline associations between sanitation, hookworm infection, anemia, stunting, and wasting.

**Methods:**

In each school, the *Schistosoma mansoni*, *S*. *haematobium*, and soil-transmitted helminth infection intensities; blood hemoglobin concentrations; heights; and weights of approximately 125 students were assessed. Of these 125 students, approximately 20 were randomly selected for student WASH surveys. Of these 20, approximately 15 were randomly selected for household sanitation observations. School WASH was also assessed through a combination of observations and questions to the headteacher. Mixed-effects logistic regression was used to compare household sanitation with hookworm infection (the other parasites being much less prevalent); and hookworm infection with anemia, stunting, and wasting.

**Findings:**

Blood, stool, and urine samples were provided by 3,729 children, and student WASH and household WASH surveys were conducted with 596 and 448 of these students, respectively.

Hookworm, *Ascaris lumbricoides*, *Trichuris trichiura*, and *S*. *mansoni* infections had prevalences of 18%, 4.8%, 0.6%, and 0.3%, respectively, and no *S*. *haematobium* infections were found. Anemia, stunting, and wasting had prevalences of 23%, 28%, and 14%, respectively.

No statistically significant associations were found between latrine absence or evidence of open defecation at home, and hookworm infection (adjusted odds ratio, OR = 1.28, 95% confidence interval, CI: 0.476–3.44; and adjusted OR = 1.21, 95% CI: 0.468–3.12; respectively); or between hookworm infection and anemia, stunting, or wasting (adjusted OR = 1.24, 95% CI: 0.988–1.57; adjusted OR = 0.992, 95% CI: 0.789–1.25; and adjusted OR = 0.969, 95% CI: 0.722–1.30; respectively).

**Conclusions:**

In this setting, no statistically significant associations were found between sanitation and hookworm; or between hookworm and anemia, stunting, or wasting. More evidence on best practices for integrated school health interventions will be gathered from the follow-up surveys in this study.

## Author summary

It is thought that inadequate sanitation may exacerbate hookworm transmission, and that hookworm infection may give rise to health problems including anemia, stunting, and wasting. Integrating monitoring of, and interventions against, these problems may yield significant cost savings. Such integrated interventions should be guided by both evidence of the relationships between the various health problems, and examples of optimally effective integration. Here, we present baseline findings from a three-year longitudinal study investigating combined school feeding; school water, sanitation, and hygiene (WASH); and deworming interventions in 30 primary schools in southern Ethiopia. In particular, we compare household sanitation, hookworm infection, and anemia, stunting, and wasting, in the schoolchildren. None of these associations were found to be statistically significant (although hookworm infection was associated with borderline statistically significantly higher odds of anemia; *P* = 0.06). The lack of significant associations may be due to the low intensities of the hookworm infections in this setting, poor conditions of latrines reducing their impact on hookworm transmission, and other factors such as malaria and inadequate diet causing anemia, stunting, and wasting.

## Introduction

Integrating school health programs might yield an efficient solution to Ethiopia’s interrelated child health problems of malnutrition [1]; soil-transmitted helminth (STH) [2, 3] and schistosome infections [3–6]; and inadequate water, sanitation, and hygiene (WASH) coverage [3, 7]. The nutritional benefits of school feeding might be reinforced with preventive chemotherapy (PC) against STHs and schistosomes, which can cause malnutrition [5, 8, 9], while malnutrition may also increase children’s susceptibility to parasitic infection [10]. Hookworm infection in particular can be an important cause of anemia, due to the gastrointestinal blood loss and decreased appetite that it can cause [8, 11], and hookworm-induced anemia can slow the cognitive and physical development of children [12]. Furthermore, the impact of PC against schistosomes and STHs might be strengthened though the improvement of WASH, if such improvements curb the parasites’ transmission.

There is increasing interest in using the school as a platform for providing various nutrition and health interventions, such as PC [13], school feeding, and health education [14]. In addition to the overlapping health benefits, combining school feeding, PC, and WASH delivery into a single platform might lead to significant cost savings [15].

A collaboration between the Ethiopian Public Health Institute (EPHI) and the Partnership for Child Development at Imperial College London, has been investigating the optimal integrated delivery of these three school health interventions, in a three-year longitudinal study in 30 government primary schools in the Southern Nations, Nationalities, and Peoples’ Region (SNNPR), Ethiopia. Here, we present baseline associations between household sanitation, hookworm infection (the other parasites being much less prevalent), and anemia, stunting, and wasting. The wider program will be used to investigate the feasibility of this form of implementation, its costs, and health and education outcomes.

## Methods

### Ethical approval and consent to participate

The study protocol was reviewed and approved by the Scientific and Ethical Review Committee (SERC) of the EPHI. On the Imperial College London side, this study was covered by ethical approval granted by the Imperial College London Research Ethics Committee for the monitoring and treatment of schistosomiasis and STHs (reference: ICREC_8_2_2). The aims and procedures of the study were explained to participants prior to enrolment in the study. Written informed consent for sample collection and student WASH surveys was obtained from the headteachers in place of the parents, and for the household WASH surveys from the heads of household. Each participant provided verbal consent, and was reminded of his or her right to withdraw from the study at any time, without consequences. Praziquantel (60 mg/kg) was administered by health officers or clinical nurses (HOCNs) to all children testing positive for schistosomiasis. In schools with non-zero prevalence of STHs, all children present were treated with 400 mg of albendazole; tablets were dispensed by the teachers, and records of the number of children treated were subsequently shared with the Regional Bureau of Health. A total of 22,258 children received PC with albendazole.

### Study area and population

The 30 schools were selected by the Ethiopian Ministry of Education in partnership with the United Nations World Food Programme (WFP). They were chosen from schools already receiving school feeding from the WFP, on the basis of suitability for home-grown school feeding; this suitability stemmed from factors such as proximity to agricultural cooperatives and local agricultural practices. Subsequently, 15 of those 30 schools were randomly selected to receive a WASH upgrade from SNV (the Netherlands Development Organisation), to enable the broader project to generate evidence for the costs and benefits of combined school health and nutrition interventions.

**Fig 1:**
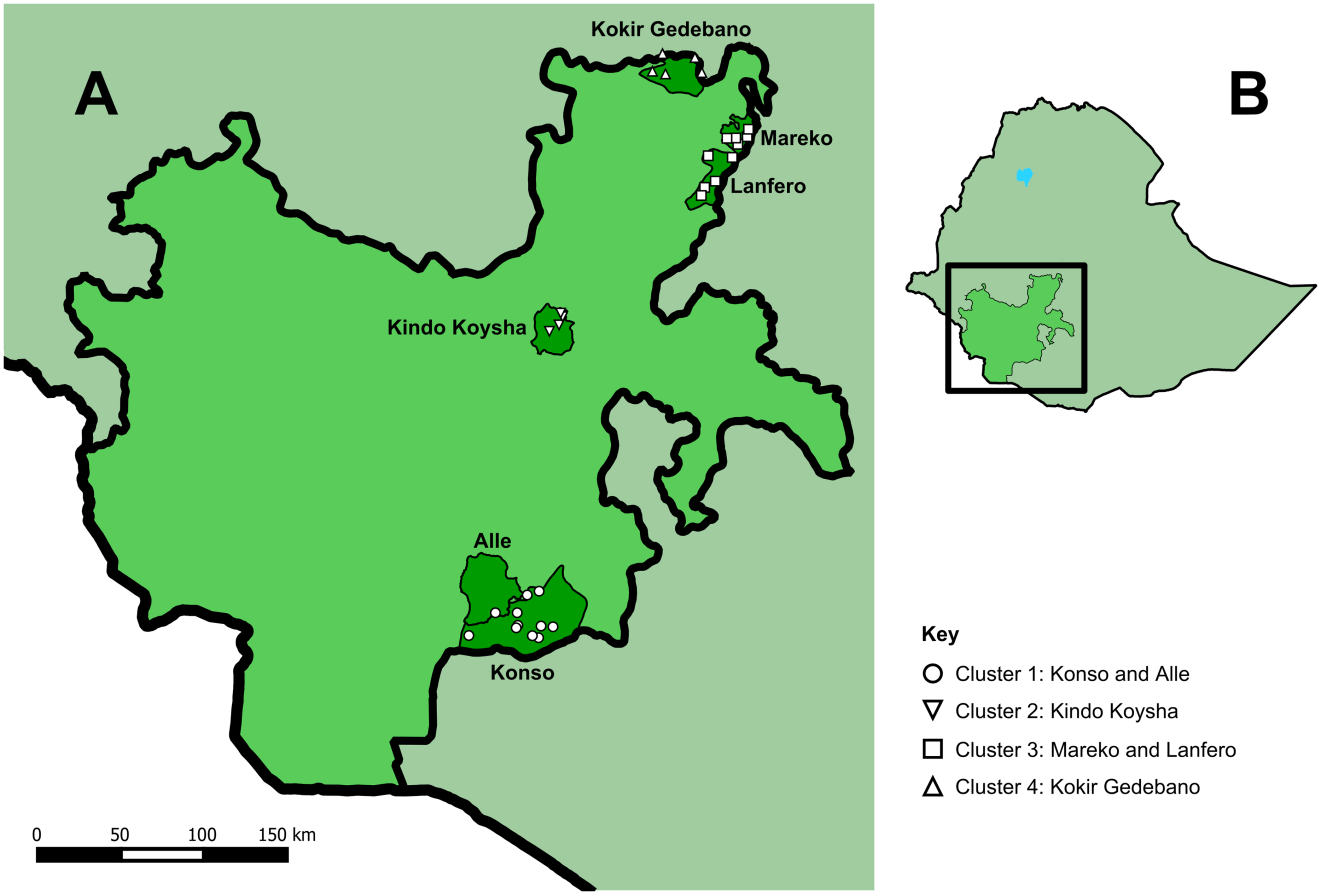
The school and woreda locations within SNNPR (A), and the position of SNNPR within Ethiopia (B). This figure was made using administrative boundaries from the GADM database of Global Administrative Areas [20].

At baseline, the schools had a combined enrolment of 30,705 students. They are distributed in four clusters, located in Konso, Alle, Kindo Koysha, Lanfero, Mareko, and Kokir Gedebano woredas, which are spread across SNNPR (Fig 1). The schools’ elevations vary from 785 m (in Konso) to 2,859 m (in Kokir Gedebano) above sea level. SNNPR has a predominantly (90.0%) rural population [16]. Mean annual rainfall in the region increases from south to north, with values between 300–1,000 mm in Konso in the south, and 1,500–3,000 mm in the north [17]. Temperature varies with elevation, but annual mean temperatures are around 20°C both in Konso, in the south, and in Jimma, which is just north of the region [18, 19].

### Survey procedures

The data were collected in June 2013 by eight teams, recruited from the zonal health offices of the included schools. Each team comprised two HOCNs and two laboratory technicians. The laboratory technicians carried out the parasitological, height, weight, and hemoglobin surveys, while the HOCNs carried out the WASH and school feeding surveys. Two days’ training prior to the survey refreshed the laboratory technicians’ understanding of the procedures to be used, familiarized the HOCNs with the survey forms to be used, and enabled standardization of the procedures across the data collectors. A third day of training included a school visit, where data was taken under close supervision and any misunderstandings were addressed.

#### School WASH

Closed-ended questionnaires were used by the HOCNs to assess school WASH infrastructure and practices. These surveys combined questions to the headteacher with observations of the condition of facilities. Schools’ positions and elevations were recorded using hand-held global positioning systems (Garmin GPS 72H, made by Garmin International, Inc., Olathe, Kansas, USA).

#### Anthropometric, hemoglobin, and parasitological surveys

Anthropometric, hemoglobin, and parasitological data were collected from approximately 125 students (63 boys and 62 girls), randomly selected (at equal intervals along lines of boys and girls) in each school. Where possible, they were selected from the grade that is two years below the final grade, to maximise the age of students and thus the risk of helminth infection [5, 9], while also allowing for two years’ follow-up of the children. Where there were not enough boys and girls in this grade, children were also randomly selected from lower grades.

The weight (in kg), height (in cm), and age (in years) of each child was recorded. Each child provided a finger prick blood sample: a sterile disposable lancet (Haemolance Plus) of 1.8 mm puncture depth was used to collect 10 μL of capillary blood into a cuvette, and a HemoCue (HemoCue A B, Ängelholm, Sweden), was used to measure the blood hemoglobin concentration.

Each child was then asked to provide one stool sample and one urine sample. Samples were immediately processed in the school. Stool samples were tested for *S*. *mansoni* and STH eggs using the Kato-Katz method [21], and urine samples were tested for hematuria, followed by filtration and microscopic examination of hematuria-positive samples for the diagnosis of *S*. *haematobium*, using the same procedures and equipment as described elsewhere [3].

#### Student WASH surveys

Of the 125 students in each school who provided stool, urine, and blood samples, 20 were randomly selected for a WASH knowledge, attitudes, and practices (KAP) survey. Wherever possible, these interviews took place away from teachers in order to minimize bias.

#### Household WASH surveys

Of the 20 students per school providing stool, urine, and blood samples, and responding to a student WASH questionnaire, 15 were randomly selected for household visits (20 and 15 having been estimated as the maximum numbers of these surveys that could be carried out while the laboratory technicians carried out the diagnoses). The HOCNs accompanied the students to their homes, where, having obtained informed consent, the HOCNs interviewed the students’ parents about household WASH practices, and inspected the household WASH facilities.

### Data management and statistical analysis

Data were collected on paper and double-entered into CS-Pro version 5 (United States Census Bureau, Washington, DC, USA). The databases were exported to SPSS version 13 (IBM Corp., Armonk, NY) for cleaning. Analyses were then conducted in R version 3.3.0 (R Foundation for Statistical Computing, Vienna, Austria).

Participants’ Kato-Katz egg counts were used to classify them as hookworm-negative (0 eggs per gram of feces, EPG), or lightly, moderately, or heavily infected in accordance with WHO guidance [22] (1–1,999, 2,000–3,999, or 4,000 EPG or more, respectively).

Children were categorized as non-, mildly, moderately, or severely anemic as defined by the WHO [23], having adjusted blood hemoglobin concentrations to account for elevation, in accordance with the same guidelines. The body mass index (BMI) was calculated for each child as the weight in kilograms divided by the square of the height in meters. Height and BMI were then transformed to height-for-age Z-score (zHFA) and BMI Z-score (zBMI), using the Box-Cox power, median and coefficient of variation (*L*, *M*, and *S*) values from the WHO Growth Reference 2007 dataset [24, 25]. Since ages were recorded in years in this study, but the WHO standards are provided for ages in months, median standard values were used for each age group in years; for example, girls of age 10 years were compared with the standards for girls of age 10 years and six months. We adopted the frequently-used convention of defining wasting and stunting as zBMI and zHFA scores of less than -2 respectively, and severe wasting and stunting as values less than -3 [26–31].

Very few hookworm infections were of moderate or heavy intensity, so light, moderate, and heavy infections were pooled to give a binary uninfected/infected variable for the subsequent comparative analysis. The anemia, stunting, and wasting variables were also condensed to binary anemic/non-anemic, stunted/non-stunted, and wasted/non-wasted variables for the comparative analysis, in order to maximize statistical power.

Overall *A*. *lumbricoides*, *S*. *haematobium*, *S*. *mansoni*, and *T*. *trichiura* prevalences were all low (4.8%, 0.0%, 0.3%, and 0.6%, respectively) compared with hookworm (18%), so while all parasitic infections were summarized, only hookworm infection was compared with sanitation and other aspects of child health. Mixed-effects logistic regressions (implemented using version 1.1–12 of the lme4 package [32]) were used to compare household sanitation risk factors (the absence of a latrine, and evidence of open defecation at home) with hookworm infection (defined as a non-zero egg count by Kato-Katz), and hookworm infection with anemia, stunting, and wasting. These regressions also accounted for age, gender, and school cluster (all as fixed effects), and school (as a random intercept). Each model excluded participants missing any relevant data, or of an age outside the range of 5–18 years.

Logistic regression’s assumption of linearity of the logit with age (the only numerical variable) was checked using the Box-Tidwell method: for each model, statistical significance (*P* < 0.05) of an introduced age*log(*age*) interaction term was taken as being indicative of a non-linear dependence on age [33–36]. The logit of wasting did exhibit such nonlinearity with age, and therefore in all models, age was split into the classes of 5–10, 11–12, and 13–18 years (chosen to maximize equality in the class sizes). Multicollinearity between the independent variables incorporated as fixed effects was assessed through the inspection of variance inflation factors (VIFs). VIFs were calculated using the vif.mer function [37], and values above two would have been taken as requiring further investigation [36, 38], but none were found.

## Results

A total of 3,729 children provided blood, stool, and urine samples, of whom 1,955 (52%) were male. Student WASH surveys were conducted with 596 of these students, and the houses of 448 were visited for WASH assessment. The 3,729 children had a mean age of 11.8 years (SD = 2.1, data missing for 21 children).

### Parasitology, anemia, and anthropometry

#### Parasitology

Of these 3,729 children, 179 were found infected with *A*. *lumbricoides* (prevalence of 4.8%), 689 with hookworm (18%), 0 with *S*. *haematobium* (0.0%), 11 with *S*. *mansoni* (0.3%), and 23 with *T*. *trichiura* (0.6%). Overall, 874 children (23%) were found positive for at least one of the above parasitic infections (481 of the boys, 25%; and 393 of the girls, 22%). Of the 689 hookworm infections, 628, 30, and 31 (91.1%, 4.4%, and 4.5%) were of light, moderate, and heavy intensity, respectively. The school-level parasitic infection prevalences and intensities are provided in S1 Table.

#### Anemia and anthropometry

Of the 3,729 children, data on blood hemoglobin concentration, age, and gender were incomplete for 40. Of the remaining 3,689, 854 (23%) were anemic. Of these 854 anemic children, 434 (51%), 386 (45%), and 34 (4.0%) were mildly, moderately, and severely anemic, respectively.

Of the 3,729 children, 21 children were missing age data, another 17 were of ages not covered by the WHO growth standards [39], and a further five were missing both weight and height data (and therefore BMI data). Of the remaining 3,686 children, 1,019 (28%) were stunted, of whom 426 (42%) were severely stunted. Of the 3,686 children, 531 (14%) were wasted, of whom 135 (25%) were severely wasted.

### School sanitation and hygiene

Of the 30 schools, 16 (53%) reported having a designated handwashing time before serving food, but only one school (3.3%) had a handwashing area away from the latrines and with piped water. All schools but one had onsite latrines, and in total there were 56 latrines in the 30 schools. These were most commonly pit latrines with cement floors (47 latrines, 84%), but six (11%) were pit latrines without cement floors, and three (5.4%) were ventilated improved pit (VIP) latrines. Eight latrine floors (14%) were cracked, one (1.8%) had collapsed completely, and one more (1.8%) was missing data, but the rest were in good structural condition. Latrine floors were most commonly described as “unclean” (30 latrines, 54%), followed by “very unclean” (22 latrines, 39%), with only four latrines (7.1%) described as “clean”. On average, there was a usable latrine stall (that is, one whose floor had not completely collapsed) for every 104 boys and one for every 109 girls. Considering only latrines with doors in addition to floors, these figures rose to 177 boys and 174 girls. Evidence of open defecation was observed in 16 of the 30 schools (53%).

### Student sanitation and hygiene

Of the 596 participants responding to student-level WASH questionnaires, the majority (388, 65%) reported no problem with school sanitation, and 434 (73%) reported no problem with home sanitation. The most common complaints were that school sanitation was too dirty (110, 18% of respondents), and that there was no toilet at home (33, 5.5% of respondents). Handwashing at various times was reported by the following numbers and proportions of students: after defecation by 299 students (50%), after urination by 116 students (19%), before eating by 526 students (88%), and when hands were visibly dirty by 467 students (78%).

### Household sanitation

Sanitation was absent in 122 (27%) of the 448 households. Of the 326 households that did have sanitation, this overwhelmingly (288, 88%) consisted of pit latrines without cement slabs. Latrines had no walls in 146 (45%) cases (data missing for two households, 0.6%), but the floors of 134 (41%) were described as “clean” (data missing for 14 households, 4.3%). Evidence of open defecation was observed at 131 (29%) households.

### Comparisons between sanitation and hookworm infection; and between hookworm infection and anemia, stunting, and wasting

#### Inadequate sanitation as a risk factor for hookworm infection

**Fig 2:**
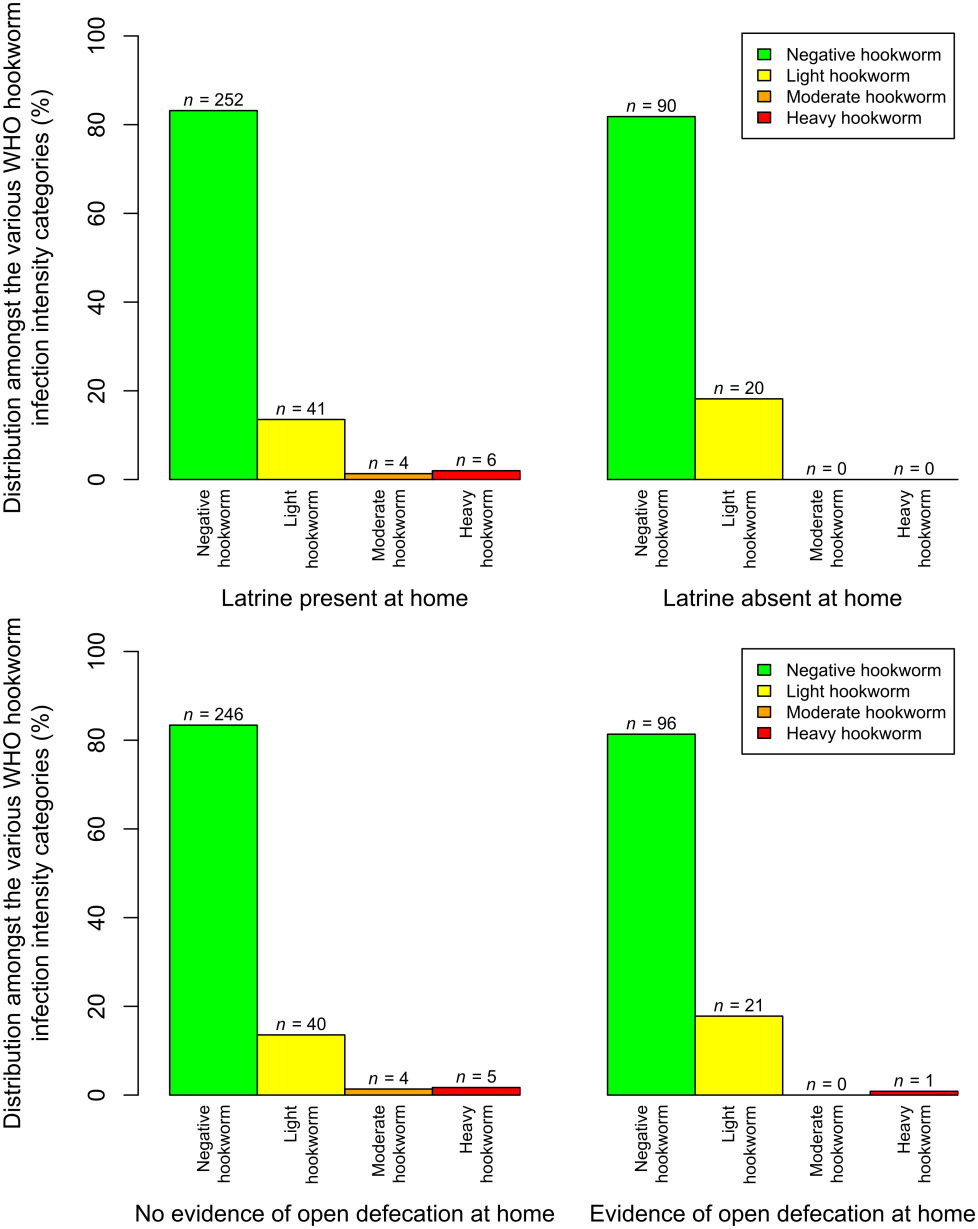
The distributions of children’s hookworm infection intensities, according to the two sanitation risk factors. Bar heights represent the percentages of the hookworm infection intensities, in children with each sanitation response. The number of children with each sanitation-hookworm infection intensity class combination is given by *n*. Light, moderate, and heavy hookworm infections were defined as 1–1,999, 2,000–3,999, and 4,000 EPG or more, respectively [22].

Fig 2 shows the distributions of children’s hookworm infection intensities, according to the two sanitation variables of latrine absence and evidence of open defecation at home. It shows how rare the heavier hookworm infection intensities were in this dataset.

Complete data on household sanitation, hookworm infection, age, gender, and school were available for 413 children. The fixed effect estimates for the model of risk factors for hookworm infection are presented in Table 1. Neither of the household sanitation variables (absence of a latrine at home, and evidence of open defecation at home: adjusted odds ratio, OR = 1.28, 95% CI: 0.476–3.44; adjusted OR = 1.21, 95% CI: 0.468–3.12; respectively) nor age or gender, were statistically significantly associated with odds of hookworm infection. Statistically significant differences were only seen between clusters (hookworm infection being statistically significantly more prevalent in clusters 2 and 3 than in cluster 1).

**Table 1:** Fixed effects from the mixed-effects logistic regression for hookworm infection. This model used data from the 413 students between the ages of 5 and 18 years, and with complete data for the included variables. Of these 413 students, 71 (17%) were positive for hookworm. The variance (in the logit scale) of the random intercept for school in this model was 1.17.

		Prevalence of this risk factor	Prevalence of hookworm infection in participants with this risk factor	Adjusted odds ratio	95% Confidence interval	*P*-value
Latrine at home?	Yes	303/413 (73%)	51/303 (17%)	Reference	-	-
	No	110/413 (27%)	20/110 (18%)	1.28	[0.476, 3.44]	0.6
Evidence of open defecation at home?	No	295/413 (71%)	49/295 (17%)	Reference	-	-
	Yes	118/413 (29%)	22/118 (19%)	1.21	[0.468, 3.12]	0.7
Gender	Female	212/413 (51%)	35/212 (17%)	Reference	-	-
	Male	201/413 (49%)	36/201 (18%)	1.15	[0.641, 2.07]	0.6
Age (years)	5 to 10	109/413 (26%)	16/109 (15%)	Reference	-	-
	11 to 12	138/413 (33%)	24/138 (17%)	1.49	[0.645, 3.43]	0.4
	13 to 18	166/413 (40%)	31/166 (19%)	1.65	[0.735, 3.69]	0.2
School cluster	1	152/413 (37%)	11/152 (7.2%)	Reference	-	-
	2	53/413 (13%)	23/53 (43%)	16.7	[3.32, 84.4]	< 0.001
	3	140/413 (34%)	31/140 (22%)	4.60	[1.26, 16.8]	0.02
	4	68/413 (16%)	6/68 (8.8%)	1.32	[0.245, 7.15]	0.7

#### Hookworm infection as a risk factor for anemia, stunting, and wasting

**Fig 3:**
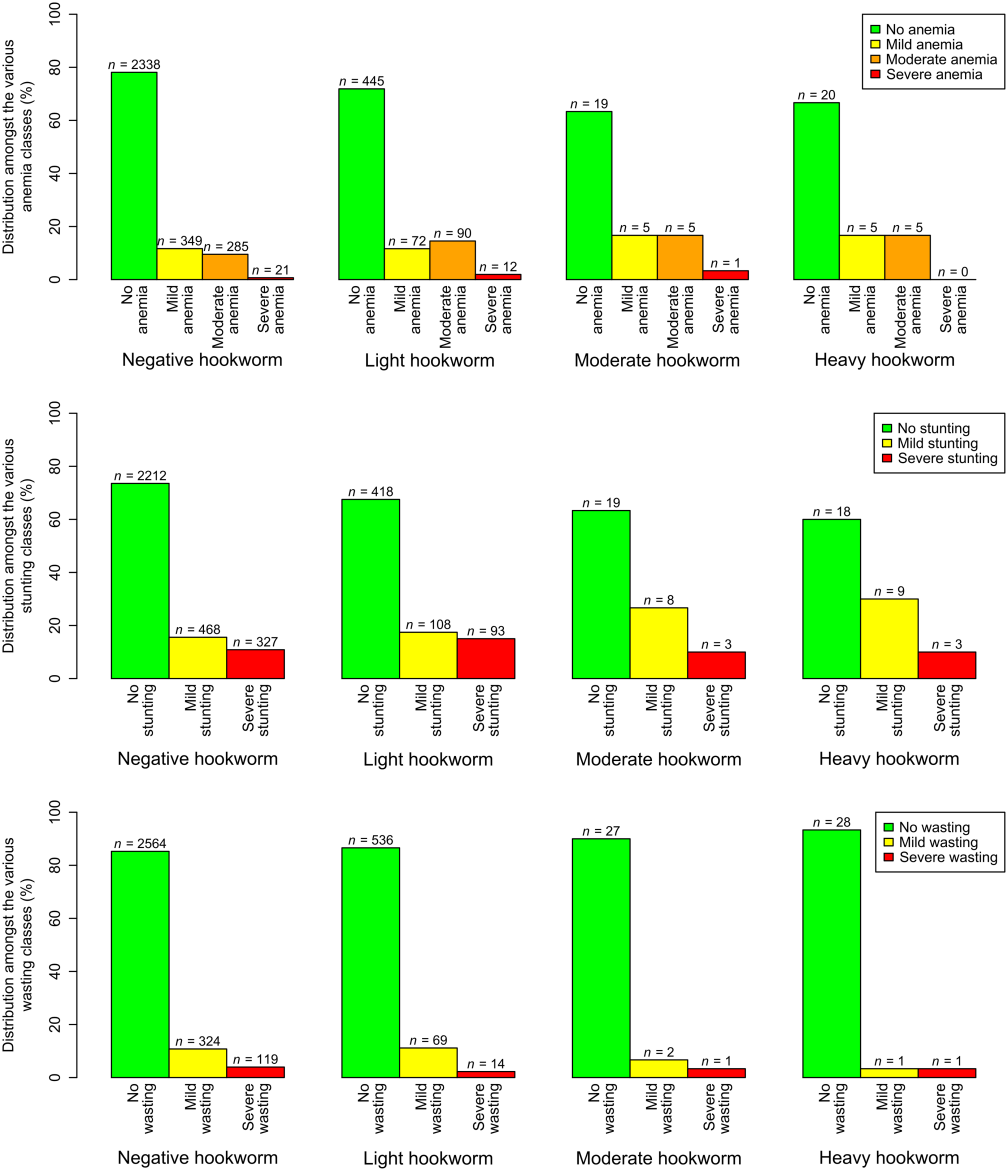
The distributions of children’s anemia, stunting, and wasting classifications according to their hookworm infection intensities. Bar heights represent the percentages of each anemia, stunting, and wasting severity, in children with each hookworm infection intensity class. The number of children with each hookworm infection intensity-anemia/stunting/wasting class combination is given by *n*. Light, moderate, and heavy hookworm infections were defined as 1–1,999, 2,000–3,999, and 4,000 EPG or more, respectively [22]. Mild stunting and wasting were defined as zHFA and zBMI values between -2 and -3, respectively, and severe stunting and wasting values below -3. Anemia classes were calculated using blood hemoglobin concentrations, taking into account age, gender, and elevation [23].

Fig 3 shows the distributions of participants’ severities of anemia, stunting, and wasting according to their hookworm infection intensities. As with Fig 2, it shows how heavier hookworm infection intensities and more severe anemia, stunting, and wasting categories were rare. Indeed, no children were found to be both heavily infected with hookworm, and severely anemic.

Complete data on hookworm infection, anemia, age, gender, and school were available for 3,672 children. The fixed effect estimates for the model of risk factors for anemia are presented in Table 2. Neither hookworm infection (adjusted OR = 1.24, 95% CI: 0.988–1.57) nor age were statistically significantly associated with odds of anemia, although the association with hookworm infection could be described as borderline significant (*P* = 0.06). Boys had a statistically significantly higher odds of anemia than girls, and compared with cluster 1, cluster 2 had significantly more anemia but clusters 3 and 4 were not significantly different.

**Table 2:** Fixed effects from the mixed-effects logistic regression for anemia. This model used data from the 3,672 students between the ages of 5 and 18 years, and with complete data for the included variables. Of these 3,672 students, 850 (23%) were anemic. The variance (in the logit scale) of the random intercept for school in this model was 0.487.

		Prevalence of this risk factor	Prevalence of anemia in participants with this risk factor	Adjusted odds ratio	95% Confidence interval	*P*-value
Hookworm infection	Negative	2993/3672 (82%)	655/2993 (22%)	Reference	-	-
	Positive	679/3672 (18%)	195/679 (29%)	1.24	[0.988, 1.57]	0.06
Gender	Female	1742/3672 (47%)	371/1742 (21%)	Reference	-	-
	Male	1930/3672 (53%)	479/1930 (25%)	1.27	[1.08, 1.49]	0.004
Age (years)	5 to 10	973/3672 (26%)	214/973 (22%)	Reference	-	-
	11 to 12	1366/3672 (37%)	305/1366 (22%)	1.13	[0.914, 1.41]	0.3
	13 to 18	1333/3672 (36%)	331/1333 (25%)	1.14	[0.906, 1.42]	0.3
School cluster	1	1345/3672 (37%)	278/1345 (21%)	Reference	-	-
	2	483/3672 (13%)	186/483 (39%)	2.57	[1.11, 5.98]	0.03
	3	1229/3672 (33%)	198/1229 (16%)	0.747	[0.393, 1.42]	0.4
	4	615/3672 (17%)	188/615 (31%)	1.87	[0.859, 4.05]	0.1

Complete data on hookworm infection, stunting, age, gender, and school were available for 3,686 children. The fixed effect estimates for the model of risk factors for stunting are presented in Table 3. Neither hookworm infection (adjusted OR = 0.992, 95% CI: 0.789–1.25) nor gender were statistically significantly associated with odds of stunting. There was a statistically significant increase in the odds of stunting with age. Compared with cluster 1, cluster 4 had significantly more stunting but clusters 2 and 3 had no significant difference.

**Table 3:** Fixed effects from the mixed-effects logistic regression for with stunting. This model used data from the 3,686 students between the ages of 5 and 18 years, and with complete data for the included variables. Of these 3,686 students, 1,019 (28%) were stunted. The variance (in the logit scale) of the random intercept for school in this model was 1.16.

		Prevalence of this risk factor	Prevalence of stunting in participants with this risk factor	Adjusted odds ratio	95% Confidence interval	*P*-value
Hookworm infection	Negative	3007/3686 (82%)	795/3007 (26%)	Reference	-	-
	Positive	679/3686 (18%)	224/679 (33%)	0.992	[0.789, 1.25]	0.9
Gender	Female	1754/3686 (48%)	469/1754 (27%)	Reference	-	-
	Male	1932/3686 (52%)	550/1932 (28%)	1.15	[0.979, 1.36]	0.09
Age (years)	5 to 10	984/3686 (27%)	209/984 (21%)	Reference	-	-
	11 to 12	1368/3686 (37%)	372/1368 (27%)	1.67	[1.32, 2.10]	< 0.001
	13 to 18	1334/3686 (36%)	438/1334 (33%)	2.10	[1.66, 2.67]	< 0.001
School cluster	1	1355/3686 (37%)	287/1355 (21%)	Reference	-	-
	2	486/3686 (13%)	159/486 (33%)	2.28	[0.646, 8.08]	0.2
	3	1230/3686 (33%)	294/1230 (24%)	1.26	[0.487, 3.26]	0.6
	4	615/3686 (17%)	279/615 (45%)	3.65	[1.14, 11.7]	0.03

Complete data on hookworm infection, wasting, age, gender, and school were available for 3,686 children. The results of the model of risk factors for wasting are presented in Table 4. Hookworm infection was not a statistically significant determinant (adjusted OR = 0.969, 95% CI: 0.722–1.30), but boys and children aged 11 years or more were at significantly higher risk. Compared with cluster 1, cluster 3 had significantly less wasting but clusters 2 and 4 were not significantly different.

**Table 4:** Fixed effects from the mixed-effects logistic regression for wasting. This model used data from the 3,686 students between the ages of 5 and 18 years, and with complete data for the included variables. Of these 3,686 students, 531 (14%) were stunted. The variance (in the logit scale) of the random intercept for school in this model was 0.381.

		Prevalence of this risk factor	Prevalence of wasting in participants with this risk factor	Adjusted odds ratio	95% Confidence interval	*P*-value
Hookworm infection	Negative	3007/3686 (82%)	443/3007 (15%)	Reference	-	-
	Positive	679/3686 (18%)	88/679 (13%)	0.969	[0.722, 1.30]	0.8
Gender	Female	1754/3686 (48%)	206/1754 (12%)	Reference	-	-
	Male	1932/3686 (52%)	325/1932 (17%)	1.54	[1.26, 1.87]	< 0.001
Age (years)	5 to 10	984/3686 (27%)	83/984 (8.4%)	Reference	-	-
	11 to 12	1368/3686 (37%)	236/1368 (17%)	2.13	[1.61, 2.82]	< 0.001
	13 to 18	1334/3686 (36%)	212/1334 (16%)	2.00	[1.49, 2.68]	< 0.001
School cluster	1	1355/3686 (37%)	255/1355 (19%)	Reference	-	-
	2	486/3686 (13%)	69/486 (14%)	0.727	[0.332, 1.59]	0.4
	3	1230/3686 (33%)	133/1230 (11%)	0.453	[0.251, 0.818]	0.009
	4	615/3686 (17%)	74/615 (12%)	0.526	[0.255, 1.08]	0.08

## Discussion

Schools have been used successfully as a platform for many different health and nutrition interventions around the world. To date, many of these interventions have been conducted in silos. In this project, the assessment of the parasitological status of students was complemented with the collection of a rich dataset pertaining to school feeding, WASH conditions of schools and households, and students’ WASH KAP. This data was collected by the same trained enumerators in a single school visit.

Of these schoolchildren, 23% were found to be infected with *S*. *mansoni* or an STH, and most of these infections were with hookworm (prevalence of 18%). This is consistent with other studies from the region. In particular, in two recent studies, Bayesian geostatistical models used climatic, socioeconomic, and parasitological survey data to predict a high prevalence of hookworm throughout much of SNNPR (and a national prevalence of 17.7%), along with much patchier distributions of *A*. *lumbricoides* and *T*. *trichiura* (and national prevalences of 8.5% and 6.1%, respectively), and very low levels of *S*. *mansoni* and *S*. *haematobium* infection (and national prevalences of 8.9% and 8.3%) [2, 6]. The high hookworm prevalence we found in Kindo Koysha aligns with the findings of a study by Taye *et al*. (2013), who, in a study of podoconiosis patients nearby, found a hookworm prevalence of 40.9% [40]. Around Butajira (a town between the Mareko and Kokir Gedebano schools), Davey *et al*. (2005) found prevalences of 16.6%, 14.7%, and 2.5% for *A*. *lumbricoides*, hookworm, and *T*. *trichiura*, respectively [41]. The prevalences of stunting and wasting that we found (28% and 14%, respectively) are comparable with the values of 45% and 9% found in a study of infants up to two years of age in Halaba (in the north of SNNPR) in 2013 [42]. Another study, in 2013 in Bule Hora, Oromia (just east of SNNPR) found prevalences of 47.6% and 13.4%, respectively, in children aged up to five years [43]. Regarding anemia, Deribew *et al*. (2010) found a prevalence of 32.4% in a study of under-fives in southern Ethiopia [44]; similar to the 23% that we found.

Hookworm transmission takes place when eggs in an infected person’s feces are deposited on the ground, before hatching to release larvae, which develop in the soil and infect a person during dermal contact with infested soil [9]. Consistent latrine use should contain these eggs and larvae, thereby reducing hookworm transmission. However, in this study, neither evidence of open defecation around the home compound, nor the absence of a latrine at home, were statistically significant predictors of hookworm infection. There are a number of possible explanations for this. Firstly, latrine presence may not lead to consistent latrine use, and although the evidence of open defecation at home was used as an indicator, this only reflects recent open defecation, while the hookworms’ lifespans have been estimated at 5–7 years [9]. Secondly, even if home sanitation has the effect of completely removing parasite eggs from soil around the household, people may still be exposed to infection away from home: indeed, it is likely that much hookworm transmission takes place between, rather than within, households [45].

These factors, along with the potential for socioeconomic confounding in observational studies (higher socioeconomic status, SES, being a cause of both better access to sanitation, and lower exposure to hookworms), may explain the mixed results of previous studies of the relationship between sanitation and hookworm infection. In a recent systematic review and meta-analysis, Strunz *et al*. (2014) did not find a statistically significant difference in odds of hookworm infection between those with and without access to sanitation [46]. Indeed, even a recent large cluster-randomized trial found no impact of sanitation on hookworm, a finding the authors attributed to insufficient coverage and use of the sanitation [47]. However, in another systematic review by Ziegelbauer *et al*. (2012), availability of sanitation was associated with a significantly lower odds of hookworm infection [48], while Ethiopia’s recent national mapping of schistosomes, STHs, and school WASH revealed a borderline statistically significant, negative association (Kendall’s *τ*_*b*_ = -0.039, 95% CI: -0.090–0.012, *P* = 0.067) between the adequacy of school sanitation and hookworm infection intensity [3]. In this survey, 88% of the latrines inspected had no cement slab. They may therefore actually be exacerbating hookworm transmission, by concentrating defecation spatially, and providing suitable conditions for larval development [49].

Participants with hookworm infections did not have statistically significantly higher odds of anemia, though the association might be described as borderline significant (*P* = 0.06). It is known that hookworms feed on their hosts’ blood, and this, as well as bleeding caused by hookworms attaching to the intestinal mucosa, can cause anemia [50, 51]. However, the impact of hookworm infection on blood hemoglobin concentration is strongly dependent on the infection intensity. A meta-analysis showed that children with heavy hookworm infections had statistically significantly lower blood hemoglobin concentrations than uninfected children, but that children with light infections did not [52]. Most (91.1%) of the hookworm infections we found were of light intensity, which may well explain the lack of a significant association between hookworm infection and anemia. Confounding by malaria and poor nutrition, both of which can also be associated with anemia [44, 53, 54], may also have weakened this association. Hookworm species were not differentiated in this study, but previous studies in Jimma (just north of SNNPR) and Mirab Abaya (in SNNPR), found the majority of hookworm infections to be with *Necator americanus*, rather than with *Ancylostoma duodenale* [55, 56]. *N*. *americanus* worms cause less blood loss than *A*. *duodenale* [12, 51]: a predominance of *N*. *americanus* worms may therefore have been another reason for the lack of an association between hookworm infection and anemia in this setting.

In all the models, statistically significant differences were seen between the clusters. As they were spread across SNNPR, there was substantial variation in elevation and climate. These climatic differences may explain some of the inter-cluster variation in all models: in the case of hookworm, a suitable environment is needed to allow development of larvae in the soil [9]. Anemia, stunting, and wasting may be caused by dietary differences, caused by climatic (as well as cultural and economic) differences between the clusters.

Hookworm-positive participants in this study did not have significantly higher odds of stunting, or wasting. This finding is supported by another study recently carried out in northern Ethiopia, which found that helminth infection (prevalence of 69.1%, 62.8% of which were hookworm infections or co-infections) was not a statistically significant determinant of either stunting or wasting [57]. A study in an area of extreme poverty in Peru, with a hookworm prevalence of 21.3% (mostly light-intensity infections), did reveal a significant association between hookworm infection and being both stunted and underweight (adjusted OR = 1.74, 95% CI: 1.05–2.86) [58]. However, no significant association between hookworm infection and stunting was found in a Brazilian community with a hookworm prevalence of 69.8% [59]. Similarly, analyses of data from the Lao People’s Democratic Republic [60], and Bolivia [61], did not reveal statistically significant associations between hookworm infection and anthropometric indicators.

Recent meta-analyses have investigated increases in height, weight, and blood hemoglobin concentration following treatment of STHs [62, 63]. These have found that mass treatment appears to have little to no effect on height or weight [62, 63], or hemoglobin [62], but that it is possible that targeted treatment of infected children does increase their weight gain, over the following six months [62]. These findings are broadly in line with ours.

In this survey, boys were at statistically significantly (*P* < 0.05) higher risk of anemia and wasting, and also at a higher risk of stunting (though statistically non-significant, *P* = 0.09). Svedberg (1990) demonstrated that boys in Sub-Saharan Africa frequently suffer worse nutrition than girls, and interpreted this as resulting from preferential treatment of girls, due to social factors such as high female participation in agricultural labor, polygamy, bridewealth (wealth transferred at marriage from the groom or his family to the bride’s parents), and early marriage of females [64]. Wamani *et al*. (2007) conducted a meta-analysis of 16 Demographic and Health Surveys in Sub-Saharan Africa, and found that boys suffered statistically significantly more stunting [65]. The authors also discussed how the cause may be biological, rather than social: boys may be more vulnerable to malnutrition because natural selection favors a sex ratio of 1:1, and the number of boys born slightly exceeds that of girls [65]. Regarding anemia, our finding that boys were at greater risk is supported by a cross-sectional survey of children aged 7–18 years in Tanzania [66].

Our analyses did not explicitly account for socioeconomic confounding, and this is perhaps their biggest drawback. Frequently-used indicators of SES, such as possession of a motorcycle or television, would have been of no use since these were completely absent in the households, but other indicators such as household floor construction, possessions at home, and ownership of livestock may have been useful. That said, most participants’ households were not visited. As recipients of WFP school feeding, these schools are all in areas considered to be food-insecure, and hence all the study participants might be considered to be of low SES.

Another limitation was the use of the Kato-Katz method on only one slide per participant, for the diagnosis of *S*. *mansoni* and STHs. This method is recognized to have a low sensitivity, particularly when slides are not examined immediately [67, 68]. It is therefore possible that some participants recorded as being hookworm-negative will in fact have had light infections.

Ideally, participants’ ages would have been recorded in months rather than years. This would have allowed for more robust comparisons with the WHO growth reference standards [25], which are provided for each month. Unfortunately, in this setting, children’s dates of birth were not available, but rather only their ages in years. Future studies that include anthropometry and interviews with parents, might benefit from the development of local calendars. Such local calendars should enable the assessment of children’s ages to within a few months. They could be developed using major events (such significant weather events including storms or droughts, or political events including elections) to ascertain the year, and then using questions about the season to estimate the month, of birth.

The student sanitation and hygiene indicators were self-reported, and shame and fear of reproach may therefore have influenced participants’ responses. For this reason, these indicators were not included in the models. Alternative approaches, such as sensors or global positioning systems (GPS) may enable the collection of more objective data, but will also be accompanied by substantial ethical and practical challenges. These include the invasions of privacy inherent in monitoring of location and WASH-related behaviors, as well as the possibility of sensors being removed.

No statistically significant associations were found between home sanitation and hookworm infection; or hookworm infection and anemia, stunting, or wasting, suggesting that in this setting, these different aspects of poor child health are not exacerbating each other. However, the lack of access to adequate sanitation, and the prevalences of hookworm, anemia, stunting, and wasting in and around these schools confirm the need for more interventions to improve child health in the region. Integrated approaches incorporating different types of interventions may prove to be the most efficient.

## Supporting information

S1 Table School details, including prevalences and mean intensities of *S*. *mansoni* and the STHs. (DOCX)
