# Supplementary material for: Sanitation, hookworm, anemia, stunting, and wasting in primary school children in southern Ethiopia: Baseline results from a study in 30 schools
Source: PLoS Negl Trop Dis. 2017 Oct 9;11(10):e0005948. doi: 10.1371/journal.pntd.0005948 (PMC5633169; doi:10.1371/journal.pntd.0005948)
Supplement: S1 Table — (DOCX) [file pntd.0005948.s001.docx]

**S1 Table: School details, including prevalences and mean intensities of *S. mansoni* and the STHs.**

| **School name** | **Cluster** | **Latitude (°N)** | **Longitude (°E)** | **Number of children tested** | **Average age (years, to 3 significant figures)** | **Prevalence of infection (%)** | | | | | **Arithmetic mean infection intensity (EPG, to 3 significant figures)** | | | |
| --- | --- | --- | --- | --- | --- | --- | --- | --- | --- | --- | --- | --- | --- | --- |
|  |  |  |  |  |  | ***S. mansoni*** | **Hookworm** | ***A. lumbricoides*** | ***T. trichiura*** | **Any of these parasites** | ***S. mansoni*** | **Hookworm** | ***A. lumbricoides*** | ***T. trichiura*** |
| Aba Roba | 1 | 5.2670 | 37.4362 | 125 | 12.3 | 0/125 (0.0%) | 0/125 (0.0%) | 2/125 (1.6%) | 0/125 (0.0%) | 2/125 (1.6%) | 0.00 | 0.00 | 48.0 | 0.00 |
| Arfaide | 1 | 5.4021 | 37.3202 | 125 | 11.7 | 0/125 (0.0%) | 12/125 (9.6%) | 0/125 (0.0%) | 0/125 (0.0%) | 12/125 (9.6%) | 0.00 | 61.1 | 0.00 | 0.00 |
| Baide | 1 | 5.4978 | 37.3746 | 122 | 8.5 | 0/122 (0.0%) | 2/122 (1.6%) | 5/122 (4.1%) | 0/122 (0.0%) | 7/122 (5.7%) | 0.00 | 3.34 | 11.6 | 0.00 |
| Eyanu | 1 | 5.4018 | 37.2018 | 125 | 12.0 | 2/125 (1.6%) | 0/125 (0.0%) | 0/125 (0.0%) | 0/125 (0.0%) | 2/125 (1.6%) | 1.73 | 0.00 | 0.00 | 0.00 |
| Fuchucha | 1 | 5.5184 | 37.4392 | 120 | 10.4 | 1/120 (0.8%) | 12/120 (10%) | 6/120 (5.0%) | 0/120 (0.0%) | 18/120 (15%) | 0.200 | 35.2 | 172 | 0.00 |
| Gera | 1 | 5.2778 | 37.4025 | 125 | 12.1 | 0/125 (0.0%) | 3/125 (2.4%) | 24/125 (19%) | 4/125 (3.2%) | 30/125 (24%) | 0.00 | 2.88 | 1400 | 1.15 |
| Gugnara Kolme | 1 | 5.3204 | 37.3149 | 125 | 12.3 | 2/125 (1.6%) | 9/125 (7.2%) | 7/125 (5.6%) | 3/125 (2.4%) | 21/125 (17%) | 3.84 | 352 | 1840 | 57.6 |
| Jarso | 1 | 5.3302 | 37.4497 | 125 | 12.2 | 0/125 (0.0%) | 0/125 (0.0%) | 0/125 (0.0%) | 0/125 (0%) | 0/125 (0.0%) | 0.00 | 0.00 | 0.00 | 0.00 |
| Kemale | 1 | 5.3338 | 37.3234 | 125 | 11.5 | 0/125 (0.0%) | 4/125 (3.2%) | 11/125 (8.8%) | 0/125 (0.0%) | 14/125 (11%) | 0.00 | 15.0 | 166 | 0.00 |
| Shekana | 1 | 5.3260 | 37.5147 | 125 | 12.1 | 1/125 (0.8%) | 4/125 (3.2%) | 6/125 (4.8%) | 1/125 (0.8%) | 12/125 (9.6%) | 0.192 | 2.88 | 4.42 | 3.46 |
| Weito | 1 | 5.2790 | 37.0577 | 125 | 10.8 | 0/125 (0.0%) | 0/125 (0.0%) | 5/125 (4.0%) | 1/125 (0.8%) | 6/125 (4.8%) | 0.00 | 0.00 | 5.95 | 0.384 |
| Fojena Mata | 2 | 6.9203 | 37.4950 | 125 | 10.9 | 0/125 (0.0%) | 77/125 (62%) | 1/125 (0.8%) | 0/125 (0.0%) | 77/125 (62%) | 0.00 | 231 | 9.60 | 0.00 |
| Mundena | 2 | 6.9546 | 37.5472 | 125 | 12.0 | 1/125 (0.8%) | 87/125 (70%) | 9/125 (7.2%) | 2/125 (1.6%) | 93/125 (74%) | 0.192 | 1680 | 1340 | 0.384 |
| Oidu Chama | 2 | 7.0184 | 37.5563 | 125 | 12.0 | 0/125 (0.0%) | 43/125 (34%) | 9/125 (7.2%) | 0/125 (0.0%) | 47/125 (38%) | 0.00 | 264 | 40.5 | 0.00 |
| Tulicha | 2 | 6.999 | 37.5671 | 125 | 13.1 | 1/125 (0.8%) | 63/125 (50%) | 15/125 (12%) | 2/125 (1.6%) | 74/125 (59%) | 1.92 | 252 | 95.2 | 4.61 |
| Edenaba Agawo | 3 | 7.6513 | 38.3197 | 125 | 11.4 | 0/125 (0.0%) | 28/125 (22%) | 8/125 (6.4%) | 0/125 (0.0%) | 36/125 (29%) | 0.00 | 74.3 | 14.8 | 0.00 |
| Elala Jirano | 3 | 8.0076 | 38.5762 | 125 | 12.4 | 0/125 (0.0%) | 61/125 (49%) | 1/125 (0.8%) | 0/125 (0.0%) | 61/125 (49%) | 0.00 | 155 | 0.192 | 0.00 |
| Faka Worabo | 3 | 7.9673 | 38.5689 | 125 | 13.2 | 0/125 (0%) | 5/125 (4.0%) | 5/125 (4.0%) | 2/125 (1.6%) | 12/125 (9.6%) | 0.00 | 1.34 | 4.42 | 0.384 |
| Goto Mandifa | 3 | 7.9590 | 38.4590 | 119 | 11.5 | 0/119 (0%) | 20/119 (17%) | 26/119 (22%) | 1/119 (0.8%) | 47/119 (39%) | 0.00 | 52.2 | 66.2 | 0.807 |
| Shirinto | 3 | 7.9266 | 38.5181 | 124 | 11.3 | 0/124 (0.0%) | 21/124 (17%) | 1/124 (0.8%) | 1/124 (0.8%) | 23/124 (19%) | 0.00 | 17.8 | 0.387 | 0.774 |
| Udasa Repi | 3 | 7.9605 | 38.5068 | 125 | 11.8 | 0/125 (0%) | 35/125 (28%) | 0/125 (0.0%) | 0/125 (0.0%) | 35/125 (28%) | 0.00 | 37.2 | 0.00 | 0.00 |
| Urgo Dubala | 3 | 7.6950 | 38.3380 | 125 | 12.4 | 0/125 (0.0%) | 28/125 (22%) | 2/125 (1.6%) | 4/125 (3.2%) | 32/125 (26%) | 0.00 | 98.5 | 2.30 | 2.69 |
| Wante Boditi | 3 | 7.7286 | 38.3959 | 125 | 12.4 | 0/125 (0.0%) | 2/125 (1.6%) | 2/125 (1.6%) | 0/125 (0.0%) | 4/125 (3.2%) | 0.00 | 0.576 | 0.384 | 0.00 |
| Wonte Sostero | 3 | 7.8561 | 38.4880 | 125 | 11.5 | 0/125 (0.0%) | 68/125 (54%) | 1/125 (0.8%) | 0/125 (0.0%) | 68/125 (54%) | 0.00 | 111 | 0.192 | 0.00 |
| Wotanbo Gobe | 3 | 7.8649 | 38.3574 | 125 | 11.6 | 0/125 (0.0%) | 32/125 (26%) | 1/125 (0.8%) | 0/125 (0.0%) | 33/125 (26%) | 0.00 | 60.9 | 0.192 | 0.00 |
| Beider | 4 | 8.3191 | 38.0544 | 125 | 11.2 | 0/125 (0.0%) | 24/125 (19%) | 0/125 (0.0%) | 0/125 (0.0%) | 24/125 (19%) | 0.00 | 14.6 | 0.00 | 0.00 |
| Combol | 4 | 8.3925 | 38.2852 | 124 | 13.4 | 0/124 (0.0%) | 18/124 (15%) | 16/124 (13%) | 0/124 (0.0%) | 32/124 (26%) | 0.00 | 29.6 | 42.0 | 0.00 |
| Gora | 4 | 8.4134 | 38.1091 | 125 | 13.4 | 2/125 (1.6%) | 26/125 (21%) | 3/125 (2.4%) | 1/125 (0.8%) | 32/125 (26%) | 1.15 | 23.8 | 0.960 | 0.192 |
| Jimma Walene | 4 | 8.3045 | 38.1252 | 125 | 11.4 | 0/125 (0.0%) | 5/125 (4.0%) | 12/125 (9.6%) | 1/125 (0.8%) | 18/125 (14%) | 0.00 | 0.960 | 5.95 | 0.384 |
| Welega Dese | 4 | 8.3117 | 38.3227 | 120 | 11.6 | 1/120 (0.8%) | 0/120 (0.0%) | 1/120 (0.8%) | 0/120 (0.0%) | 2/120 (1.7%) | 0.200 | 0.00 | 0.400 | 0.00 |
| Overall: | - | - | - | 3729 | 11.8 | 11/3729 (0.3%) | 689/3729 (18%) | 179/3729 (4.8%) | 23/3729 (0.6%) | 874/3729 (23%) | 0.315 | 120 | 177 | 2.44 |
